# Seoul Virus Infection and Subsequent Guillain-Barré Syndrome in Traveler Returning to France from Kenya, 2022

**DOI:** 10.3201/eid3102.241387

**Published:** 2025-02

**Authors:** Tristan M. Lepage, Charlotte Boullé, Vincent Le Moing, Vincent Foulongne, Virginie Sauvage

**Affiliations:** Centre Hospitalier Universitaire de Montpellier, Montpellier, France (T.M. Lepage, C. Boullé, V. Le Moing, V. Foulongne); Institut de Recherche pour le Développement, Montpellier (T.M. Lepage, C. Boullé, V. Le Moing); Institut Pasteur, Paris, France (V. Sauvage); Université Paris Cité, Paris (V. Sauvage)

**Keywords:** Seoul virus, orthohantavirus, viruses, hantavirus, hemorrhagic fever with renal syndrome, Guillain-Barré syndrome, Kenya, Africa, zoonoses, rat-borne diseases, vector-borne infections, France

## Abstract

Seoul virus (SEOV) is a worldwide ratborne orthohantavirus. We describe an SEOV infection in an adult returning to France from Kenya, followed by Guillain-Barré syndrome. We confirmed SEOV infection by PCR and sequencing. Although transmission might have occurred in Kenya, the epidemiologic information available is not sufficient to confirm that possibility.

Seoul virus (SEOV), an orthohantavirus member of the Hantaviridae family, was first identified in South Korea in 1982 (*1*). Hosted in brown rats (*Rattus norvegicus*), SEOV has been isolated multiple times on 4 continents (Asia, Europe, America, and Africa) because of the worldwide distribution of its host (*2*). SEOV can cause mild to moderate hemorrhagic fever with renal syndrome (HFRS), which has a case-fatality rate of ≈1%–2% (*3*). Transmission to humans occurs through inhalation of aerosolized rodent excreta (saliva, urine, or feces) or through direct contacts and bites (*4*). Virologically confirmed SEOV human infections have been reported in America, Europe, and Asia, but not yet in Africa, despite virus detection in rats in Senegal (*5*) and Benin (*6*) and serologic hints of its circulation in humans (*2*,*7*).

We report an unexpected SEOV infection in an adult returning from Kenya. The infection was complicated by Guillain-Barré syndrome (GBS), a rare condition associated with hantavirus infections (*8*).

## Case Report

A 54-year-old man with a remote history of mitral and tricuspid valve repair caused by Barlow’s disease sought treatment at an emergency department in August 2022 in Montpellier, France. He had a 6-day history of fever up to 40°C and had chills and generalized myalgias. The night before admission, he had a single episode of hematemesis. Two weeks before, he had returned from a 5-day business trip in Nairobi, Kenya. He visited warehouses and stayed in urban areas and reported having no freshwater baths, animal contact, or unprotected sex. He was not taking any medications.

At admission, the patient was afebrile (37.8°C) and hemodynamically stable. Physical examination was unremarkable, indicating no mucocutaneous petechiae or any other signs of bleeding. Laboratory results were notable for elevated hepatic transaminases (aspartate aminotransferase 335 U/L [reference range <40 U/L], alanine aminotransferase 347 U/L [reference range <41 U/L]), unremarkable prothrombin time and bilirubin, elevated serum creatinine (324 µM [reference range 59–104 μM]) with proteinuria (protein-to-creatinine ratio 240 mg/mmol [reference range <23 mg/mmol]), increased C-reactive protein (70.5 mg/L [reference range <5 mg/L]), and thrombocytopenia (54,000 platelets/µL [reference range 150,000–400,000 platelets/µL]). Peripheral leukocyte counts were within reference ranges. Results of a chest radiograph followed by an abdominal computed tomography with contrast were unremarkable. Blood and urine cultures were sterile. A *Plasmodium*-specific PCR result was negative. Serologic tests for hepatitis viruses, HIV, cytomegalovirus, *Coxiella*, *Rickettsia*, and *Bartonella* were negative, whereas *Toxoplasma* and Epstein-Barr virus serologic tests were consistent with past infections. PCR and serologic tests for arboviruses (dengue, chikungunya, Zika, West Nile, yellow fever, and Rift Valley fever virus) and *Leptospira* were negative.

We hydrated the patient intravenously and administered intravenous ceftriaxone (2 g/d) for presumed leptospirosis, which was stopped 4 days after admission. He remained afebrile during his stay and had no recurrent bleeding. Levels of serum creatinine (218 µM), aspartate aminotransferase (93 UI/L), alanine aminotransferase (187 UI/L), and platelets (327,000/µL) improved, and the patient was discharged on day 5 of hospitalization.

Three days after discharge, the patient’s hantavirus-specific IgM and IgG serologic tests returned positive results (Hantavirus Mosaic 1 IgM and IgG indirect immunofluorescence assay; EUROIMMUN, https://www.euroimmun.com). As is usual in France, for surveillance purposes, we transferred the positive sample to the National Reference Centre for Hantavirus, which confirmed the result by ELISA and indirect immunofluorescence. We then identified SEOV in the patient’s serum sample (collected on day 6 after symptom onset) by real-time reverse transcription PCR (cycle threshold 34.37) (*9*). We obtained a complete SEOV small segment sequence and partial medium and large segment sequences by using an in-house amplicon-based nanopore sequencing method (GenBank accession nos. PQ241127–9). We performed a phylogenetic analysis of the complete coding sequence of the small segment (Figure).

**Figure:**
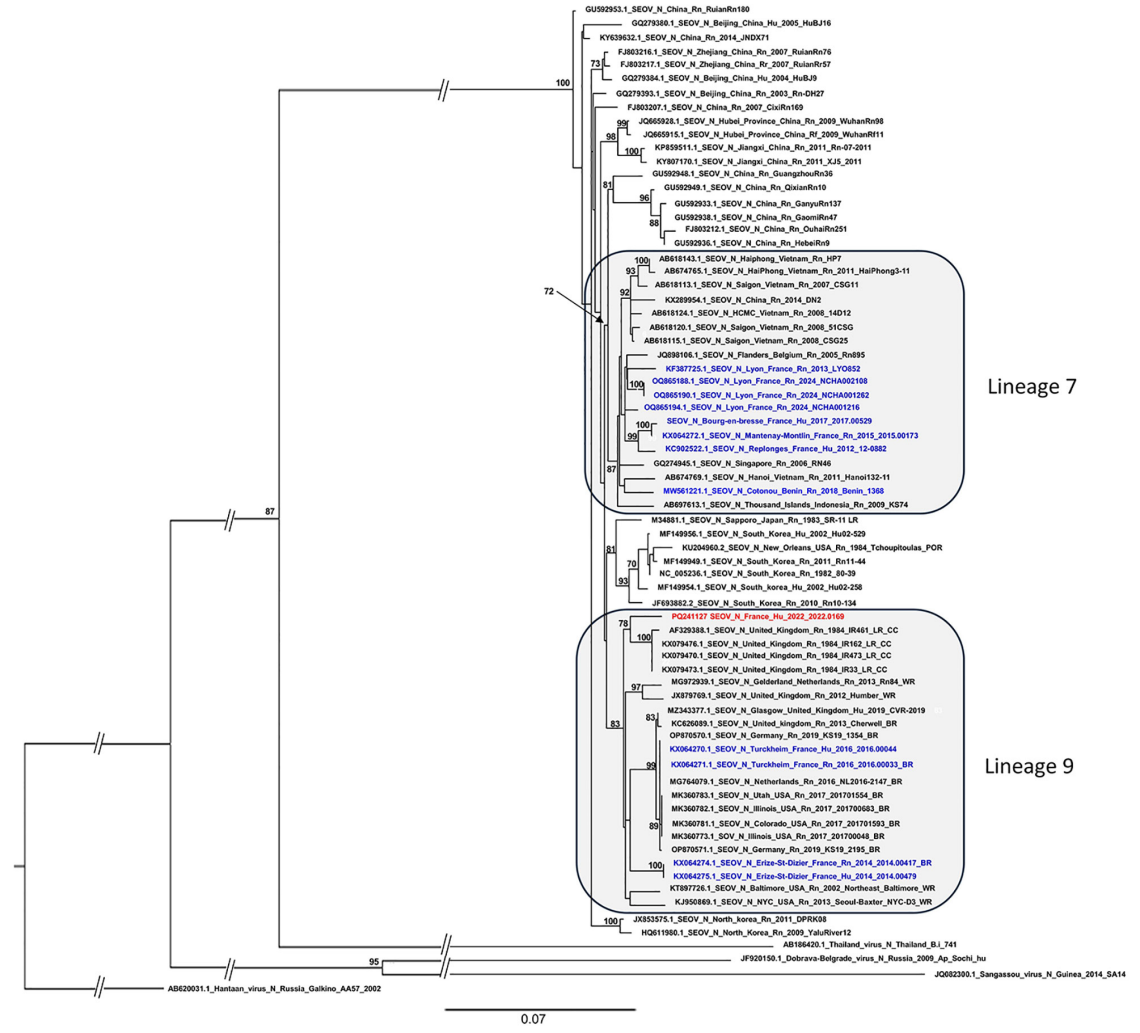
Phylogenetic tree based on the complete coding region (1,290 bp) of the small segment of SEOV strain detected in an infected patient in France, 2022, and representative strains of SEOV and other hantavirus species. The complete segment Bayesian tree was reconstructed using MAFFT version 7.023b (https://mafft.cbrc.jp/alignment/software) and RAxML 8.2 (https://cme.h-its.org/exelixis/web/software/raxml) with the general time-reversible plus gamma distribution substitution model and a rapid bootstrap (i.e., general time-reversible invariable site plus discrete Gamma model, bootstraps = 1,000). The numbers at each node are bootstrap probabilities (>70%) as determined for 1,000 iterations. The SEOV strain Hu_2022_2022.0169 (GenBank accession no. PQ241127) retrieved in this study is indicated in red, whereas other sequences from France and Benin are represented in blue. GenBank accession numbers are provided for reference viruses. Hantaan virus was used as outgroup. Scale bars indicate nucleotide substitutions per site. BR, breeder rat (includes feeder and pet rats); CC, cell culture; Hu, human; LR, laboratory rat; N, nucleoprotein; Rn, *Rattus norvegicus*; Rr, *R. rattus*; SEOV, Seoul virus; WR, wild rat.

Five days after his discharge, the patient was admitted to the neurology department because of progressive ascending paresthesia and unsteady gait that manifested 10 days after symptom onset. On physical examination, he had ataxia with a positive Romberg test, diffuse areflexia, apallesthesia, and paresthesia of both lower legs. He had no motor deficit or respiratory failure, and cranial nerve testing was unremarkable. Cerebrospinal fluid analysis was notable for albuminocytologic dissociation (proteinorachia 1.48 g/L [reference range <0.4 g/L]), a negative meningitis multiplex PCR panel, and sterile culture. Electromyoneurographic analysis revealed demyelinating motor neuropathy and non–length dependent sensory neuropathy. Results of a spinal cord magnetic resonance imaging were unremarkable, and antiganglioside antibody test results were negative. We made a diagnosis of GBS and treated the patient with intravenous immunoglobulins (2 g/kg for 5 d). His neurologic impairments gradually improved, and the patient was discharged 5 days after admission. Complete resolution of disease ensued ≈90 days after symptom onset.

## Conclusions

We identified SEOV in an adult with moderate HFRS, which is the 10th confirmed SEOV infection in France since SEOV was isolated in a human in 2012 (*10*). Unlike previous cases, this infection occurred in a traveler returning from Kenya, where he had visited warehouses. Although no signs of rodent presence were reported, transmission might have occurred through aerosolized rodent excreta. Symptom onset occurred 13 days after arrival in Kenya, which is consistent with transmission occurring in Kenya, given SEOV’s known incubation period of ≈2–3 weeks (*3*).

Until the early 2000s, evidence of hantavirus circulation in Africa relied solely on seroprevalence studies in small mammals and humans (*11*,*12*), which were prone to cross-reactivity and lacked confirmatory assays. In 2006, Sangassou virus was isolated from mice in Guinea, constituting the first virologic evidence of hantaviruses in Africa (*13*). Further investigations then revealed several novel hantaviruses across the entire continent in rodents, shrews, and bats (*14*). SEOV strains were identified in black rats in Senegal (*5*) and in brown rats in Benin (*6*), confirming circulation in West Africa. To date, only a few human hantavirus infections have been described in Africa on the basis of serologic evidence (*15*), although amplification of hantavirus virologic material from human samples would provide more specific proof of infection.

Phylogenetic analysis of the complete small segment coding sequence of this patient’s SEOV strain (Figure) showed that it belongs to lineage 9 (as partial segments of medium and large sequences). This result was unexpected, given that lineage 9 strains have mostly been identified in pet, feeder, and laboratory rats (from Europe and the United States), whereas the patient reported no contact with such rats. In comparison, SEOV sequences isolated in Benin belonged to lineage 7, whereas those isolated in Senegal were only partial, belonging to lineages 3 and 4.

We acknowledge that the patient might also have been infected in France, potentially through exposure to rodent excreta while handling boxes in his garage days before traveling to Kenya, although no signs of rodent presence were reported. However, given the absence of lineage 9 circulation data in wild rats both in France and Africa, we cannot confirm either hypothesis.

Ten days after symptom onset, SEOV infection was followed by GBS, which fully resolved after immunoglobulin administration. To date, 6 cases of hantavirus-related GBS have been reported in Europe and China since 1992 (*8*), occurring 1–2 weeks after the onset of HFRS. However, hantavirus diagnoses were made on the basis of serologic evidence alone, limiting further virus characterization. Whether the risk for GBS varies according to the hantavirus species involved remains unclear.

In conclusion, SEOV can cause moderate infections but might also lead to severe complications such as GBS. Greater awareness of SEOV among healthcare providers and policymakers is essential to improve access to testing and enhance data on global circulation.
